# EXPLORING TRANSITIONAL CARE UNITS IN ONTARIO CANADA: A QUALITATIVE STUDY

**DOI:** 10.1093/geroni/igae098.3973

**Published:** 2024-12-31

**Authors:** Katherine McGilton, Alexandra Krassikova, Lydia Yeung, Sandra McKay, Martine Puts, Sara Guilcher, Kerry Kuluski, Jennifer Bethell

**Affiliations:** Toronto Rehabilitation Institute – University Health Network, Toronto, Ontario, Canada; KITE Research Institute, Toronto, Ontario, Canada; KITE Research Institute, Toronto, Ontario, Canada; VHA Home HealthCare, Toronto, Ontario, Canada; University of Toronto, Toronto, Ontario, Canada; University of Toronto, Toronto, Ontario, Canada; University of Toronto, Toronto, Ontario, Canada; Toronto Rehabilitation Institute – University Health Network, Toronto, Ontario, Canada

## Abstract

Transitional care units (TCUs) provide short-term, low-intensity, restorative care to older adults who are medically fit to leave the hospital but cannot do so due to various factors, such as the availability of home care support or long-term care beds. TCUs are similar to skilled nursing facilities in the US, also referred to as intermediate care models, post-acute care or alternate care units depending on the country of origin. In Ontario, Canada, TCUs were introduced in the past decade, and little is known about them. The objectives of this study were to examine the processes of care of TCUs in Ontario employing an exploratory qualitative design. Semi-structured interviews were conducted with seven TCU managers. A five-step inductive thematic analysis adapted from Braun and Clarke was applied to the data to identify themes. Interviewees were on average 43 years of age; four identified as men and three as women. Years of experience as a TCU manager at the time of the interview ranged from 2 months to 5 years. Four themes were identified: a) going above and beyond; 2) managing patients’ expectations; 3) creating a team that works together; 4) navigating a constantly changing environment. Qualitative findings demonstrate the important role of TCUs in helping patients transition from acute care to other settings. TCUs are filling in a gap in the healthcare system of Ontario and need to be recognized as a core service. Qualitative findings may be used by healthcare organizations and unit managers to guide the operation of TCUs.

